# Effects of apolipoprotein A5 haplotypes on the ratio of triglyceride to high-density lipoprotein cholesterol and the risk for metabolic syndrome in Koreans

**DOI:** 10.1186/1476-511X-13-45

**Published:** 2014-03-12

**Authors:** Seongwon Cha, Hyunjoo Yu, Ah Yeon Park, Kwang Hoon Song

**Affiliations:** 1 KM Health Technology Research Group, Medical Research Division, Korea Institute of Oriental Medicine, 1672 Yuseongdae-ro, Yuseong-gu, Daejeon 305-811, Republic of Korea; 2 SME Partnership Center, Korea Institute of Oriental Medicine, 1672 Yuseongdae-ro, Yuseong-gu, Daejeon 305-811, Republic of Korea

**Keywords:** APOA5, ZNF259, Haplotype, Triglceride-to-HDL cholesterol ratio, Triglyceride, HDL cholesterol, Metabolic syndrome, Polymorphism

## Abstract

**Background:**

Single-nucleotide polymorphisms (SNPs) around the apolipoprotein A5 gene (*APOA5*) have pleiotropic effects on the levels of triglyceride (TG) and high-density lipoprotein cholesterol (HDL-C). *APOA5* SNPs have also been associated with metabolic syndrome (MS). Here, we constructed haplotypes with SNPs spanning *APOA5* and *ZNF259*, which are approximately 1.3 kb apart, to perform association analyses with the risk for MS and the levels of TG and HDL-C in terms of a TG:HDL-C ratio.

**Methods:**

The effects of three constructed haplotypes (TAA, CGG, and CGA, in the order of rs662799, rs651821, and rs6589566) on the TG:HDL-C ratio and MS were estimated using multiple regression analyses in 2,949 Koreans and in each gender separately (1,082 men and 1,867 women).

**Results:**

The haplotypes, CGG and CGA, were associated with the TG:HDL-C ratio and the risk of MS development in both genders. That is, the minor alleles of the rs662799 and rs651821 in *APOA5,* irrespective of which allele was present at rs6589566, had the marked effects. Interestingly, a C–G–A haplotype at these three SNPs had the most marked effects on the TG:HDL-C ratio and the risk of MS development in women.

**Conclusions:**

We have identified the novel *APOA5-ZNF259* haplotype manifesting sex-dependent effects on elevation of the TG:HDL-C ratio as well as the increased risk for MS.

## Introduction

Single-nucleotide polymorphisms (SNPs) in the apolipoprotein A5 gene (*APOA5*), located on chromosome 11q23 near the *APOA1–C3–A4* gene cluster, have pleiotropic effects on changes in the levels of triglycerides (TG) and high-density lipoprotein cholesterol (HDL-C) [1,2]. Recently, a genome-wide association study (GWAS) reported that the minor allele of a SNP, rs964184, has a bivariate effect on TG-elevation and HDL-C-decrease [3]. These pleiotropic bivariate associations of *APOA5* SNPs, in turn, affect the risk for metabolic syndrome (MS) [1,2,4].

Haplotype analyses of association of *APOA5* SNPs with lipid levels and risk for MS have focused primarily on variants located within *APOA5* or within the *APOA1–C3–A4–A5* gene cluster [5-12]. In particular, the *APOA5* haplotypes, APOA5*1, and *2, and *3, comprised of rs662799 (-1131T>C), rs3135506 (56C>G), rs2072560 (IVS3 + 476G>A), and rs2266788 (1259T>C), have been studied for association with lipid levels and MS [5,11-13]. The APOA5*2 haplotype, determined by the minor alleles of rs662799, rs2072560, and rs2266788, has been associated with elevated TG levels and risk for the development of MS [11-13], while the APOA5*3 haplotype, determined by the minor allele of rs3135506, has been shown to associate with increased levels of TG [4,5].

However, *APOA5* is adjacent to the zinc finger protein 259 gene (*ZNF259*; approximately 1.3 kb downstream of *APOA5*), so that separation of the effects of these closely located genes is difficult. In fact, it is possible that crosstalk between *APOA5* and *ZNF259* contributes to modulation of plasma TG levels. Although the precise mechanisms associated with this have yet to be determined, research has indicated that APOA5 interaction with proteoglycan-bound lipoprotein lipase and with low-density lipoprotein receptor can reduce plasma TG levels by hydrolysis of TG-rich lipoproteins and by endocytosis of remnant lipoproteins, respectively [14]. *ZNF259* encodes a zinc finger protein ZPR1 that binds to the cytoplasmic tyrosine kinase domain of epidermal growth factor receptor (EGFR) in quiescent cells [15]. When mitogens such as EGF disrupt ZPR1 binding by tyrosine phosphorylation of EGFR, ZPR1 is translocated to the nucleus. It has been reported that remnant lipoproteins, among TG-rich lipoproteins, induce EGFR phosphorylation in smooth muscle cells, which results in smooth muscle cell proliferation and atherosclerosis risk [16] and that EGF concentrations in plasma and peripheral blood mononuclear cells associate with lipid concentrations, including TG and HDL-C [17]. Furthermore, the variants exerting effects on TG and HDL-C levels, including rs662799, rs3135506, rs651821, rs2072560, rs2266788, rs6589566, and rs964184, cluster together and are in strong linkage disequilibrium (LD) with each other around the two genes [3,18,19]. Therefore, construction of haplotypes with variants spanning both genes is necessary to estimate the precise TG-elevating and HDL-C-lowering effects of *APOA5* SNPs in a population.

The current study aimed to determine the effects of haplotypes constructed from variants located around *APOA5* and *ZNF259* on the levels of TG and HDL-C, in terms of a ratio of TG to HDL-C, as well as on the risk for MS development. Due to the stronger effects of TG levels on risk of coronary disease in the context of HDL-C levels [20] and the high correlation between the levels of TG and HDL-C, the TG:HDL-C ratio can be a more efficient predictor of the risk for coronary artery diseases [21,22]. In addition, sex-dependent association patterns of these haplotypes were evaluated due to the reported gender effect of *APOA5* haplotypes on MS risk [11].

## Results

### Characteristics of recruited subjects and constructed haplotypes

**Figure 1:**
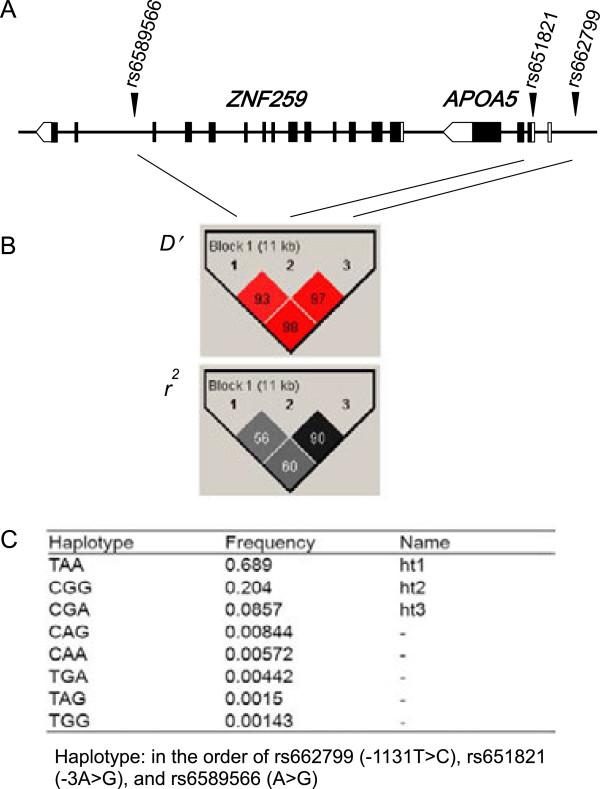
**Genetic location, linkage disequilibrium (LD), and haplotypes of single-nucleotide polymorphisms (SNPs). (A)** The SNPs rs662799 and rs651821 are respectively located in the promoter and 5′ untranslated regions of *APOA5* that has 4 exons, whereas SNP rs6589566 is found in intron 12 of *ZNF259* that has 14 exons. **(B)** LD structure of three SNPs: *D′* and *r*^*2*^. **(C)** Haplotype sequence and frequency: haplotypes with frequencies over 5% were named as ht1, ht2, and ht3.

Characteristics of the recruited subjects are presented in Table 1 and Additional file 1. Age and the prevalence of MS between men and women, as well as the gender proportion according to MS status, were not statistically different, unlike other clinical characteristics. As the linkage of rs6589566 (A>G) with rs662799 (-1131T>C; *r*^*2*^ = 0.60) and rs651821 (-3A>G; *r*^*2*^ = 0.56) in the studied population was weaker than that in the Asian HapMap population (both *r*^*2*^ = 0.80), three rather than two *APOA5–ZNF259* haplotypes with an allele frequency > 5% were constructed, such that TAA (ht1: 68.9%), CGG (ht2: 20.4%), and CGA (ht3: 8.57%) in the order of rs662799, rs651821, and rs6589566 (Figure 1). The genotypes of the three SNPs did not deviate from Hardy–Weinberg equilibrium (*p* > 0.05). Therefore, the genetic effects of these three haplotypes on a ratio of log[TG] (log-transformed TG): HDL-C and MS risk were evaluated in Koreans.

**Table 1:** Characteristics of recruited subjects

**Characteristic**	**All (n = 2,949)**	**Men (n = 1,082)**	**Women (n = 1,867)**	***P*** *
Age (y)	48.20 ± 15.73	47.87 ± 15.84	48.39 ± 15.66	0.628
BMI (kg/m^2^)	23.50 ± 3.339	24.06 ± 3.219	23.17 ± 3.364	<0.0001
WC (cm)	84.19 ± 9.890	87.20 ± 8.867	82.45 ± 10.03	<0.0001
SBP (mmHg)	119.9 ± 15.72	123.3 ± 14.86	117.9 ± 15.88	<0.0001
DBP (mmHg)	77.12 ± 11.26	79.38 ± 11.08	75.80 ± 11.16	<0.0001
FBG (mg/dL)	98.91 ± 28.24	102.2 ± 29.92	97.00 ± 27.05	<0.0001
TG (mg/dL)	126.8 ± 82.83	148.1 ± 95.48	114.4 ± 71.70	<0.0001
HDL-C (mg/dL)	47.16 ± 12.35	42.58 ± 10.81	49.81 ± 12.41	<0.0001
Log[TG]:HDL-C	0.04672 ± 0.01553	0.05303 ± 0.01622	0.04306 ± 0.01386	<0.0001
MS (%)	32.18	33.46	31.44	0.276

Values are indicated as mean ± standard deviation.

*Abbreviations: BMI* body mass index, *WC* waist circumference, *SBP* systolic blood pressure, *DBP* diastolic blood pressure, *FBG* fasting blood glucose, *TG* triglyceride, *HDL-C* high-density lipoprotein cholesterol, *Log[TG]:HDL-C* log-transformed TG:HDL-C ratio, *MS* metabolic syndrome.

*Wilcoxon rank sum test except chi-squared test for MS.

### Associations of *APOA5-ZNF259* haplotypes with TG:HDL-C ratio and MS risk

Carriage of haplotypes ht2 or ht3, versus the ht1 haplotype, was significantly associated with increased levels of the log[TG]: HDL-C ratio (for ht2 vs ht1, effect = 0.00222, 95% CI = 0.00130 - 0.00315; for ht3 vs ht1, effect = 0.00460, 95% CI = 0.00313 - 0.00607), whereas carriage of the ht3 haplotype versus the ht2 haplotype showed only a tendency toward elevating the log[TG]:HDL-C ratio (Table 2). When the population was stratified by gender, the genetic effects of the two haplotypes, ht2 and ht3, versus ht1 haplotype on log[TG]:HDL-C ratio remained significant, in both genders (for ht2 vs ht1: effect = 0.00328, 95% CI = 0.00154 - 0.00502, in men and effect = 0.00157, 95% CI = 0.00507 - 0.00264 in women; for ht3 vs ht1: effect = 0.00606, 95% CI = 0.00324 - 0.00888 in men and effect = 0.00391, 95% CI = 0.00223 - 0.00558 in women). Interestingly, haplotype ht3, with reference to haplotype ht2, was significantly associated with elevation of the log[TG]:HDL-C ratio only in women (effect = 0.00473, 95% CI = 0.00130 - 0.00816). Therefore, the ht3 haplotype showed a sex-dependent association with the log[TG]:HDL-C ratio.

**Table 2:** **Association of haplotypes in *APOA5 *** **and *ZNF259 *** **gene regions with log[TG]:HDL-C ratio**

**Subject**	**Hap 1 & 2**	**11/12/22 [n]**	**11/12/22 [mean (sd)]**	Effect (95% CI)*	***P*** *
All	ht1, ht2	1,398/838/115	0.0448 (0.0145)/0.0471 (0.0155)/0.0509 (0.0165)	0.00222 (0.00130 - 0.00315)	2.68E-06
	ht1, ht3	1,398/340/17	0.0448 (0.0145)/0.0491 (0.0173)/0.0587 (0.0154)	0.00460 (0.00313 - 0.00607)	1.05E-09
	ht2, ht3	115/123/17	0.0509 (0.0165)/0.0537 (0.0180)/0.0587 (0.0154)	0.00289 (-0.000221 - 0.00599)	0.0686
Men	ht1, ht2	482/325/42	0.0508 (0.0155)/0.0528 (0.0156)/0.0609 (0.0159)	0.00328 (0.00154 - 0.00502)	2.25E-04
	ht1, ht3	482/124/5	0.0508 (0.0155)/0.0567 (0.0176)/0.0590 (0.0125)	0.00606 (0.00324 - 0.00888)	2.83E-05
	ht2, ht3	42/54/5	0.0609 (0.0159)/0.0605 (0.0194)/0.0590 (0.0125)	0.00122 (-0.00486 - 0.00731)	0.690
Women	ht1, ht2	916/513/73	0.0417 (0.0128)/0.0435 (0.0143)/0.0452 (0.0140)	0.00157 (0.000507 - 0.00264)	3.84E-03
	ht1, ht3	916/216/12	0.0417 (0.0128)/0.0447 (0.0156)/0.0585 (0.0170)	0.00391 (0.00223 - 0.00558)	5.20E-06
	ht2, ht3	73/69/12	0.0452 (0.0140)/0.0484 (0.0150)/0.0585 (0.0170)	0.00473 (0.00130 - 0.00816)	7.16E-03

*The effect (beta), 95% confidence interval (CI), and *p*-value for Hap 2 versus Hap 1 were estimated by linear regression, after adjusting for age, sex (in all subjects), physical activity (three categories), daily food intake (three categories), clinical history, and medications for hypertension, dyslipidemia, and/or diabetes.

The sex-dependent association of the haplotype ht3 was more marked in analyses of the risk of MS (Table 3). The genetic effect of the ht2 haplotype versus the ht1 haplotype on an increased risk for MS remained significant in men (odds ratio (OR) = 1.50, 95% CI = 1.16 – 1.94), while the effect of ht3 haplotype versus ht1 haplotype remained significant in women (OR = 1.41, 95% CI = 1.00 – 1.97). Moreover, association of the ht3 haplotype versus the ht2 haplotype was significant only in women (OR = 2.67, 95% CI = 1.25 – 6.07). Taken together, unlike men, women harboring the ht3 haplotype were the most susceptible to MS risk and elevation of the log[TG]:HDL-C.

**Table 3:** **Association of haplotypes in *APOA5 *** **and *ZNF259 *** **gene regions with metabolic syndrome (MS) risk**

**Subject**	**Hap 1 & 2**	Subgroup*	**11/12/22 [n (%)]**	**OR (95% CI)**^**†**^	***P***^**†**^
All	ht1, ht2	Control	988 (61.3)/558 (34.6)/66 (4.09)	1	
		Case	410 (55.5)/280 (37.9)/49 (6.63)	1.33 (1.13 - 1.57)	0.000529
	ht1, ht3	Control	988 (80.8)/229 (18.7)/6 (0.491)	1	
		Case	410 (77.1)/111 (20.9)/11 (2.07)	1.41 (1.09 - 1.82)	0.00906
	ht2, ht3	Control	66 (45.5)/73 (50.3)/6 (4.14)	1	
		Case	49 (44.5)/50 (45.5)/11 (10.0)	1.31 (0.799 - 2.17)	0.285
Men	ht1, ht2	Control	336 (58.9)/215 (37.7)/19 (3.33)	1	
		Case	146 (52.3)/110 (39.4)/23 (8.24)	1.50 (1.16 - 1.94)	0.00197
	ht1, ht3	Control	336 (80.2)/81 (19.3)/2 (0.477)	1	
		Case	146 (76.0)/43 (22.4)/3 (1.56)	1.43 (0.950 - 2.14)	0.0842
	ht2, ht3	Control	19 (34.5)/34 (61.8)/2 (3.64)	1	
		Case	23 (50.0)/20 (43.5)/3 (6.52)	0.766 (0.356 - 1.63)	0.489
Women	ht1, ht2	Control	652 (62.6)/343 (32.9)/47 (4.51)	1	
		Case	264 (57.4)/170 (37.0)/26 (5.65)	1.21 (0.976 - 1.50)	0.0813
	ht1, ht3	Control	652 (81.1)/148 (18.4)/4 (0.498)	1	
		Case	264 (77.6)/68 (20.0)/8 (2.35)	1.41 (1.00 - 1.97)	0.0463
	ht2, ht3	Control	47 (52.2)/39 (43.3)/4 (4.44)	1	
		Case	26 (40.6)/30 (46.9)/8 (12.5)	2.67 (1.25 - 6.07)	0.0142

*Control: subjects with <3 MS risk factors ((1) abdominal obesity with waist circumference ≥ 90 cm for men and ≥ 80 cm for women, (2) systolic blood pressure ≥ 130 mmHg, diastolic blood pressure ≥ 85 mmHg, or medication for hypertension, (3) TG ≥ 150 mg/dL, (4) HDL-C < 40 mg/dL for men and < 50 mg/dL for women, and (5) fasting blood glucose ≥ 110 mg/dL or medication for hyperglycemia); case: subjects with ≥3 MS risk factors.

^†^The odds ratio (OR), 95% confidence interval (CI), and *p*-value for Hap 2 versus Hap 1 were estimated by logistic regression, after adjusting for age, sex (in all subjects), physical activity (three categories), daily food intake (three categories), clinical history, and medications for hypertension, dyslipidemia, and/or diabetes.

## Discussion

Recent GWAS findings indicated that genes with variants influencing MS per se included the *APOA5*, *ZNF259*, and *BUD13 homolog*); these genes are known to play an important role in lipid metabolism [2]. We also recently reported that the *APOA5* - 1131T>C polymorphism (rs662799) is associated with the risk of MS because of its marked effect on serum TG and HDL-C levels in Korean subjects [1]. The current study has afresh identified that haplotype constructed by SNPs in the closely positioned *APOA5–ZNF259* gene region was sex-dependently associated with the ratio of TG to HDL-C and the risk of MS development in Koreans. Specifically, haplotype ht3, viz., C–G–A at rs662799, rs651821, and rs6589566, respectively, was most significantly associated with elevation of the log[TG]:HDL-C ratio, as well as MS susceptibility, in women. We suggest that a causal variant in LD with this *APOA5*–*ZNF259* haplotype may give rise to TG and HDL-C variations and thus contribute to the risk of MS and coronary artery disease.

In terms of sex-specific influences of the *APOA5*–*ZNF259* haplotype on the TG:HDL-C ratio and MS risk, rs6589566, which is located in the *ZNF256* intron, will have an important role only in women, such that the major allele of the *ZNF256* SNP in combination with the minor alleles of the two *APOA5* SNPs can result in greater susceptibility to elevation of the TG:HDL-C ratio and MS risk (Tables 2 and 3). In contrast, in men, the minor alleles of the two SNPs located in the *APOA5* locus (rs662799 and rs651821), rather than the rs6589566 allele, will produce the dominant effects. In other words, sex-dependent association of the *APOA5* SNPs with the TG:HDL-C ratio and MS risk can be determined by using haplotype analyses of the SNPs in the *APOA5–ZNF259* locus. These gender effects are different from those previously reported [11], although we also found greater susceptibility to MS with the presence of the ht2 haplotype in men than in women (Table 3), which is similar to the previously reported gender effect of the APOA5*2 haplotype on MS risk [11].

In a study involving haplotype analysis of a 150-kb region spanning the apolipoprotein gene cluster (*APOA1–C3–A4–A5*), the SNPs in *ZNF259* were separated from those in the *APOA5* haplotype block [6]. However, in this haplotype analysis, rs1942478 and rs603446 in the *ZNF259* intron, were weakly correlated with rs6589566, which is in strong LD with *APOA5* SNPs: *r*^*2*^ = 0.125 for rs1942478 and *r*^*2*^ = 0.164 for rs603446 in the Chinese HapMap population [6]. Therefore, the *APOA5* haplotype block can be extended to the *ZNF259* gene region using rs6589566 and rs964184 located in the *ZNF259* intron.

The most obvious weakness of our study is that it is cross-sectional; thus, it would be necessary to confirm the sex-dependent effects of the *APOA5–ZNF259* haplotypes on cardiometabolic risk via longitudinal analyses. Although haplotype ht3 and the TG:HDL-C ratio appear to function in identifying cardiometabolic risk, we cannot conclude that the TG:HDL-C ratio will be as effective in predicting clinical outcomes as a diagnosis of MS. However, despite these weaknesses, the simplicity of the TG:HDL-C ratio approach has obvious advantages: the TG:HDL-C ratio, which relies on two commonly used laboratory measurements, can provide a relatively simple way to identify apparently healthy insulin-resistant persons with increased cardiometabolic risk, since the TG:HDL-C concentration ratio is significantly related to a direct measure of insulin-mediated glucose disposal [23-25].

## Conclusions

In conclusion, we found that haplotypes formed from three SNPs located in *APOA5* and *ZNF259*, viz., rs662799, rs651821, and rs6589566, were significantly associated with susceptibility to MS and elevation of the log[TG]:HDL-C ratio, sex-dependently, in Koreans; in particular, the ht3 haplotype was associated with these traits in women. We believe that our findings will help to further develop the clinical utility of the *APOA5* haplotypes and the TG:HDL-C ratio as a way to identify cardiometabolic risk.

## Methods

### Study population

All subjects provided written informed consent to participate in the study, and the study was approved by the Institutional Review Board of the Korea Institute of Oriental Medicine.

We recruited 2,949 subjects from 22 oriental medical clinics who were a part of the Korea Constitution Multicenter Study for 6 years since 2006 [1,26]. This population contained 690 patients who had hypertension, dyslipidemia, and/or diabetes. None of the subjects had a history of cancer treatment, thyroid dysfunction, or postmenopausal hormonal therapy. The subjects with MS were defined according to the modified guidelines of National Cholesterol Education Program Adult Treatment Panel III (NCEP ATP III) [27], which stipulated that at least three of the following five criteria had to be met: (1) abdominal obesity with waist circumference ≥ 90 cm for men and ≥ 80 cm for women [28], (2) systolic blood pressure ≥ 130 mmHg, diastolic blood pressure ≥ 85 mmHg, or medication for hypertension, (3) TG ≥ 150 mg/dL, (4) HDL-C < 40 mg/dL for men and < 50 mg/dL for women, and (5) fasting blood glucose ≥ 110 mg/dL or medication for hyperglycemia.

### Genotyping of SNPs

Since TG-elevating and HDL-C-lowering SNPs in *APOA5* and *ZNF259* (rs662799, rs651821, rs2072560, rs2266788, rs6589566, and rs964184) are strongly correlated with each other in Asian HapMap population (*r*^*2*^ > 0.8), we selected three SNPs across the two genes, viz., rs662799 (-1131 of *APOA5*; one of representative SNPs for APOA5*2 haplotype), rs651821 (-3 of *APOA5*; a coding SNP in the 5′ untranslated region and one of the representative SNPs for APOA5*2 haplotype), and rs6589566 (a SNP in *ZNF259* intron 12). For example, rs6589566 was strongly linked both with rs964184 (*D′* = 0.98, *r*^*2*^ = 0.97 in Asian HapMap population) and with rs2266788 (*D′* = 1.0, *r*^*2*^ = 0.98 in Asian HapMap population). The rs3135506 was not selected as a representative SNP for APOA5*3 haplotype, as it is monomorphic in Asian populations.

The genotypes of the three SNPs were determined using two genotyping methods, by using the Affymetrix Genome-Wide Human SNP array 5.0 (Affymetrix, Santa Clara, CA) and unlabeled oligonucleotide probes (UOPs) on the given polymorphic nucleotides [29]. The Affymetrix SNP array 5.0 was used for rs6589566 in 882 subjects [30], and the UOP genotyping method was used for the three SNPs (in the rest of the 2,067 subjects for rs6589566). The details of the genotyping method using UOPs (oligonucleotide sequences: Additional file 2) have been described in a previous report [1].

### Statistical analysis

The chi-squared test was used to determine whether the SNPs deviated from Hardy–Weinberg equilibrium in studied population. LD (Lewontin’s *D′ = D/|D*_*max*_*|* and *r*^*2*^) was obtained from Haploview program, version 4.2 (Daly Lab at the Broad Institute, Cambridge, MA) [31]. Construction of haplotypes of *APOA5* SNPs was implemented with Phase, version 2.1, which is a Bayesian algorithm-based program [32,33]. Multiple linear regression analyses were performed for a ratio of log[TG] to HDL-C, after adjusting for age, sex, physical activity (three categories), daily food intake (three categories), clinical history of hypertension, dyslipidemia, and/or diabetes, and medications for the three diseases. Multiple logistic regression analyses with the same adjustments, except medications, used in the multiple linear regressions were used for determining the effects of SNPs on MS risk. The *p*-values of < 0.05 were considered significant. All statistical analyses were performed using R, version 2.15.2 (http://www.r-project.org/).

## Abbreviations

APOA5: Apolipoprotein A5; 95% CI: 95% confidence interval; GWAS: Genome-wide association study; HDL-C: High-density lipoprotein cholesterol; MS: Metabolic syndrome; OR: Odds ratio; SNP: Single-nucleotide polymorphism; log[TG]: Log-transformed triglyceride; ZNF259: Zinc finger protein 259.

## Competing interests

The authors declare that they have no competing interests.

## Authors’ contributions

SC conceived and designed the study, performed the statistical analyses, interpreted the data, and drafted the manuscript. HY acquired the data, performed the statistical analyses, and helped to draft the manuscript. AYP acquired the data and helped to draft the manuscript. KHS conceived and designed the study, interpreted the data, and drafted the manuscript. All authors approved the final manuscript.

## Supplementary Material

Additional file 1 Characteristics of the subjects with MS and control subjects. Click here for file

Additional file 2 **The oligonucleotide sequences of primers and UOPs for 3 SNPs of *APOA5 *** **genotyped in Koreans.** Click here for file
